# The humerus of *Eusthenopteron*: a puzzling organization presaging the establishment of tetrapod limb bone marrow

**DOI:** 10.1098/rspb.2014.0299

**Published:** 2014-05-07

**Authors:** S. Sanchez, P. Tafforeau, P. E. Ahlberg

**Affiliations:** 1 Department of Physiology and Developmental Biology, Uppsala University, Norbyvägen 18A, Uppsala 752 36, Sweden; 2 European Synchrotron Radiation Facility, BP220, 6 rue Jules Horowitz, Grenoble Cedex 38043, France

**Keywords:** early tetrapod limb bone, growth plate, evolution, synchrotron virtual bone histology, life history

## Abstract

Because of its close relationship to tetrapods, *Eusthenopteron* is an important taxon for understanding the establishment of the tetrapod body plan. Notably, it is one of the earliest sarcopterygians in which the humerus of the pectoral fin skeleton is preserved. The microanatomical and histological organization of this humerus provides important data for understanding the evolutionary steps that built up the distinctive architecture of tetrapod limb bones. Previous histological studies showed that *Eusthenopteron*'s long-bone organization was established through typical tetrapod ossification modalities. Based on a three-dimensional reconstruction of the inner microstructure of *Eusthenopteron*'s humerus, obtained from propagation phase-contrast X-ray synchrotron microtomography, we are now able to show that, despite ossification mechanisms and growth patterns similar to those of tetrapods, it also retains plesiomorphic characters such as a large medullary cavity, partly resulting from the perichondral ossification around a large cartilaginous bud as in actinopterygians. It also exhibits a distinctive tubular organization of bone-marrow processes. The connection between these processes and epiphyseal structures highlights their close functional relationship, suggesting that either bone marrow played a crucial role in the long-bone elongation processes or that trabecular bone resulting from the erosion of hypertrophied cartilage created a microenvironment for haematopoietic stem cell niches.

## 1. Introduction

The Tetrapoda, predominantly terrestrial vertebrates with limbs rather than paired fins, are the most adaptively divergent group among the Sarcopterygii and arguably among the Osteichthyes as a whole. Extant tetrapods form a well-defined clade distinguished from their closest living relatives (the lungfishes) by numerous synapomorphies affecting all aspects of their biology. These synapomorphies must have arisen within the tetrapod stem group between the last common ancestor of tetrapods and lungfishes (where the tetrapod total group originated) and the last common ancestor of extant amphibians and amniotes (the tetrapod crown-group node). Many of the ‘key characters’ of tetrapods (e.g. limbs with digits, sacrum, fenestra ovalis, hyomandibula modified as stapes [1]) first appear over a relatively short segment of the stem group, approximately between the nodes subtending *Tiktaalik* and *Acanthostega* [2,3], and thus presumably evolved rapidly and in concert, but the ‘fish–tetrapod transition’ as a whole was a protracted process. Tetrapods that are unambiguously fully terrestrial do not appear in the fossil record until the Viséan (late Early Carboniferous), some 60 Myr after the oldest trackways with digits [4,5].

While fossils from the lower and upper ends of the tetrapod stem group are similar to other extant lobe-finned ‘fishes’ and to crown-group tetrapods, respectively, the middle segment of the stem group contains taxa with combinations of tetrapod synapomorphies and plesiomorphic characteristics that are not seen in any living vertebrate. Apomorphies relating to soft anatomy, physiology and behaviour were also being acquired step by step [6–8], but unfortunately we have very limited direct evidence for these changes.

One of the few palaeobiological data sources available to us is the microanatomy and histology of the bones. The limb bones are of particular interest here because of their functional role in the transition from water to land. The major elements of the paired appendage endoskeleton are conserved throughout the tetrapod stem and crown group, and can for the most part be homologized with endoskeletal fin elements in extant lungfishes and coelacanths [9–12]. Detailed homologies with the elements of actinopterygian fin skeletons are more difficult to establish, but the overall homology of the skeletons is uncontroversial [9,13]. However, patterns of growth and ossification in the appendage endoskeletons differ greatly between tetrapods and actinopterygians [14,15], as does the occurrence and nature of bone marrow. These differences probably reflect evolutionary innovations in the tetrapod stem group.

The few studies that have investigated the histology of fin skeletons of tetrapod stem group members [16–18] have all focused on *Eusthenopteron*, a relatively crownward form closely related to tetrapods [2]. Although Meunier & Laurin [17] concluded that tetrapod-like mechanisms of ossification already existed in *Eusthenopteron* long bones, Laurin *et al*. [16] noted that the compactness profile at mid-shaft was different from extant aquatic tetrapods and assumed that it would be characteristic of the primitively aquatic condition of *Eusthenopteron*. All these studies were based on two-dimensional examination of thin sections, a destructive technique that yields limited datasets because of the need to conserve the rare and precious specimens of fossil appendage bones.

Here, we present a non-destructive three-dimensional approach for a new microanatomical and palaeohistological analysis of stem tetrapods in an ontogenetic framework, using propagation phase-contrast X-ray synchrotron microtomography. We were able to image multiple specimens of the primitively aquatic sarcopterygian *Eusthenopteron* (juvenile and adult bones) to produce a more comprehensive palaeobiological dataset and draw more detailed conclusions than was hitherto possible. Subsequent papers will examine members of the tetrapod stem group with more derived character states, in order to cast light on the biology of the terrestrialization process and the evolution of tetrapod limbs.

## 2. Material and methods

**Figure 1.**
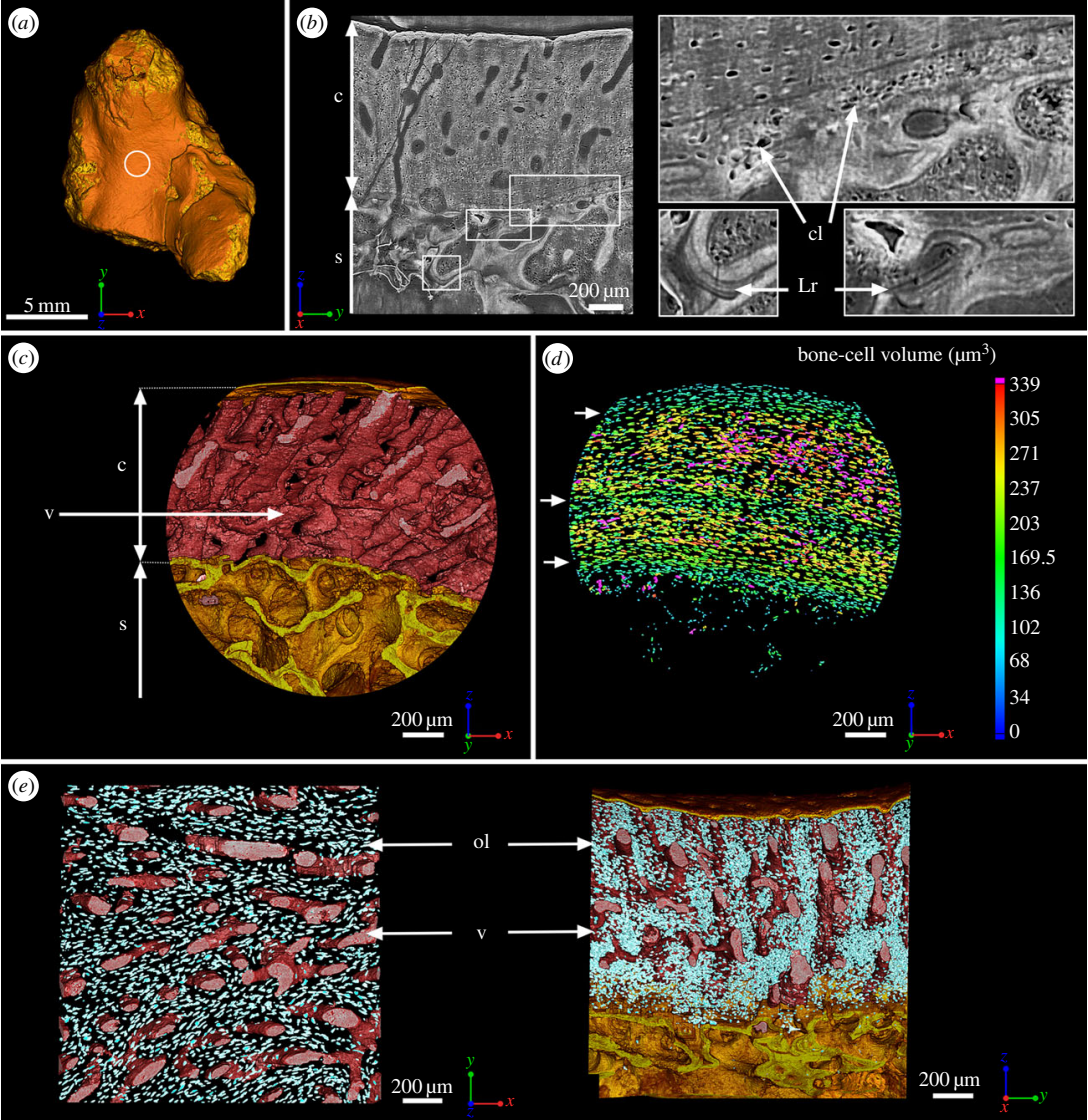
Mid-shaft bone histology of the juvenile humerus of *Eusthenopteron* (NRM P246c). (*a*) Mesial view of the whole humerus showing the location of the high-resolution scan made at mid-shaft (voxel size: 0.678 μm). (*b*) Virtual thin section (made along the longitudinal axis) showing the primary bone deposit of cortical bone and its connection to the spongiosa. Some remnants of calcified cartilage are still preserved at the location of Katschenko's line (chondrocyte lacunae) and within the spongiosa (Liesegang rings). (*c*) Transverse view of the three-dimensional organization of the vascular mesh embedded within the cortical bone and the underlying trabecular spongiosa. (*d*) Quantification of the volume of bone cells showing three recurrent periods of volume decrease (green layers pointed out with white arrows) interpreted as phases of decreased growth. (*e*) From left to right: top and longitudinal views of the vascular mesh showing the circular and radial alignment of the vascular canals (in pink). The osteocyte lacunae are represented in bright blue. c, cortical bone; cl, chondrocyte lacunae; Lr, Liesegang rings; ol, osteocyte lacunae; s, spongiosa; v, vascular mesh.

**Figure 2.**
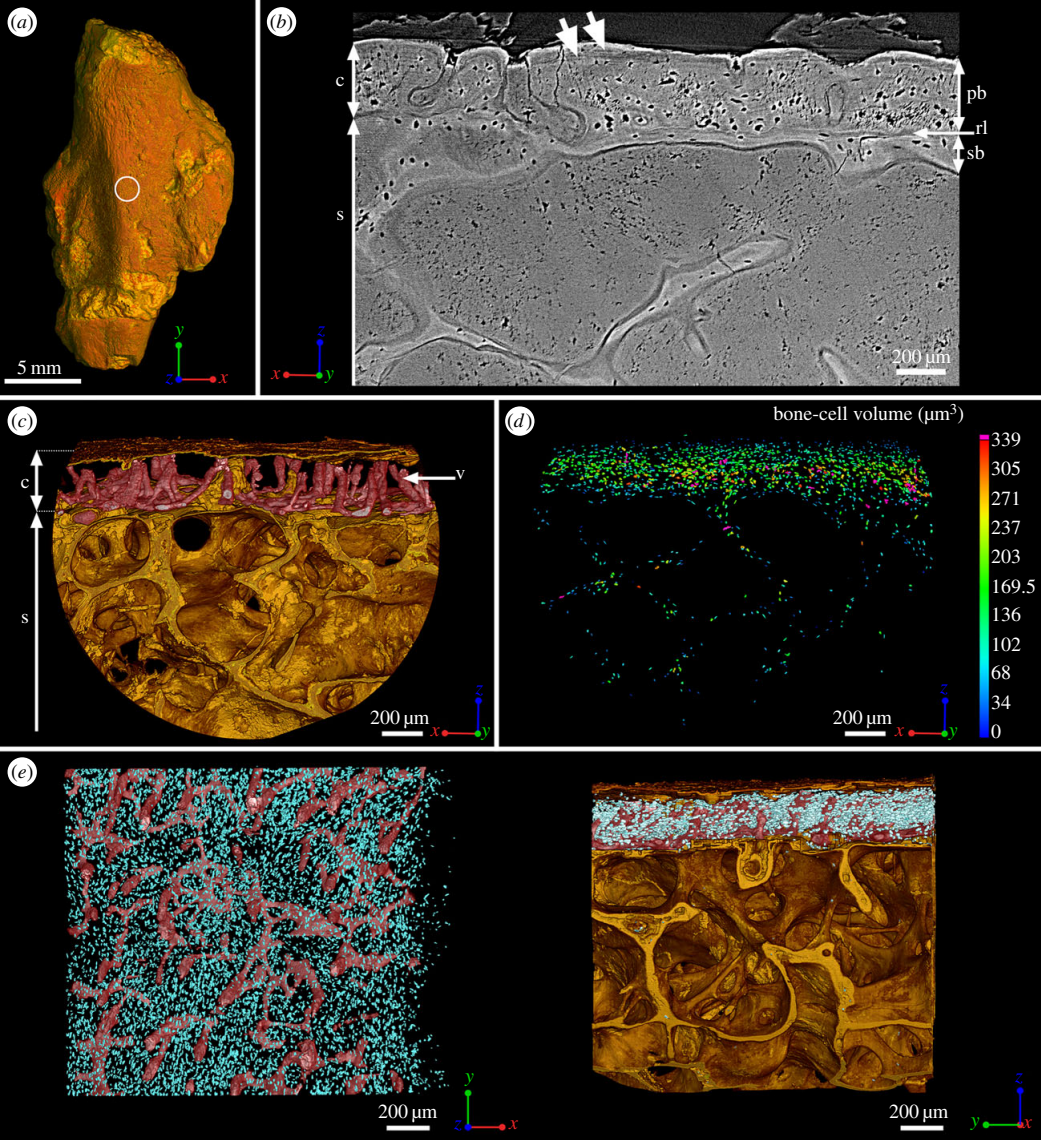
Mid-shaft bone histology of the adult humerus of *Eusthenopteron* (NRM P248d). (*a*) Mesial view of the whole humerus showing the location of the high-resolution scan made at mid-shaft (voxel size: 0.678 μm). (*b*) Transverse virtual thin section showing the primary bone deposit of cortical bone, the innermost part of which has been drastically eroded. Although X-ray tomography does not allow the nature of the bone matrix (i.e. the collagen fibre organization) to be determined, secondary bone can be distinguished from primary cortical bone because it is always demarcated by a resorption line. A secondary bone deposit of cellular endosteal bone is laid down on the inner surface of the primary cortex. The trabeculae of the spongiosa are also covered by a thin layer of cellular endosteal bone. (*c*) Transverse view of the three-dimensional organization of the vascular mesh embedded within the cortical bone and the underlying trabecular spongiosa. (*d*) Quantification of the volume of bone cells showing an obvious decrease of volume just under the surface of the primary bone and in the endosteal bone (blue) (cf. electronic supplementary material, figure S8 for detail). (*e*) From left to right: top and longitudinal views of the vascular mesh showing a non-oriented organization of the vascular canals (same colour code as for figure 1). c, cortical bone; pb, primary bone; rl, resorption line; s, spongiosa; sb, secondary bone; v, vascular mesh.

*Eusthenopteron* occurs abundantly at the 380 Myr-old locality of Miguasha, Quebec, Canada (Frasnian, Late Devonian [19]). The abundance of fossil material makes it possible to investigate its ontogeny by means of size series that can be taken as approximate representations of growth series. We focused on the three-dimensionally preserved humeri of one small and two large individuals of *Eusthenopteron* from the collection of Naturhistoriska Riksmuseet in Stockholm. The small humerus (NRM P246c) is incompletely ossified and is interpreted as juvenile (figure 1*a*). One of the large humeri (NRM P248d) is preserved in articulation with the proximal end of the ulna (figure 2*a*) and is associated with more distal elements of the fin endoskeleton as well as the proximal ends of lepidotrichia. These elements were also scanned for comparative purposes.

The specimens were imaged using propagation phase-contrast X-ray synchrotron radiation microtomography (PPC-SRμCT) at beamline ID19, European Synchrotron Radiation Facility (ESRF, Grenoble, France). A multiscale approach [20,21] was applied from 20.24 to 0.678 μm (see the electronic supplementary material for technical details).

A phase retrieval approach, based on a homogeneity assumption, was employed for reconstructing the data, using a modified version [21] of the algorithm developed by Paganin *et al*. [22]. Virtual thin sections were made using the protocol established by Tafforeau and Smith for virtual histology of teeth [20,23].

## 3. Results

### (a) Juvenile humerus

A transverse virtual thin section taken at mid-shaft in the juvenile humerus exhibits an extensive spongiosa (88% of the section area) surrounded by a 650–850 µm-thick layer of compact cortical bone (electronic supplementary material, figure S1). The spongiosa consists of numerous endochondral bone trabeculae, averaging 80 µm in thickness, that are densely and homogeneously distributed. A longitudinal thin section of the humerus shows several longitudinal tubular spaces within the trabecular mesh crossing the whole bone from the proximal epiphysis towards one of the distal epiphyses (electronic supplementary material, figure S1*b*).

At mid-shaft (figure 1*a*), the inner surface of the cortical bone is delimited by clusters of numerous large globular cell lacunae (cl, figure 1*b*,*d*) that can be identified as chondrocyte lacunae of cartilage. This suggests that remnants of Katschenko's line [14,24–26] are still present. Several stacks of Liesegang rings [24], typical of calcified cartilage, are also notable among the endochondral trabeculae (Lr, figure 1*b*). In extant tetrapods, the spongiosa forms when chondroclasts create erosion bays in the cartilage that are then lined with a thin peripheral bone layer, and it is common for small remnants of calcified cartilage to be left behind by the process; spongiosa formation in *Eusthenopteron* appears to have been similar.

The compact cortical bone exhibits a uniform primary tissue (figure 1*b*) with numerous flattened osteocyte lacunae, ranging in volume between 100 and 340 µm^3^ (figure 1*d*). It contains two complete and one partial cycle of progressively increasing osteocyte volumes, each complete cycle measuring 350–450 µm in thickness (figure 1*d*). The bone cell lacunae are mostly aligned in parallel with the peripheral surface of the bone. They are evenly organized around a dense vascular mesh. These canals are obliquely radial and parallel with each other. They average 42 µm in diameter (figure 1*c*,*e*).

Towards the epiphysis (electronic supplementary material, figure S2*a*), the metaphyseal compact cortical bone, separated from the spongiosa by a cementing line, is made of primary bone tissue pierced with a dense vascularization (vc, electronic supplementary material, figure S2*b*) surrounded by numerous flattened osteocyte lacunae (ol, electronic supplementary material, figure S2*b*). The metaphyseal region shows numerous extrinsic fibres embedded in the bone matrix (ef, electronic supplementary material, figure S2*b*). The proximal ends of these fibres are cut off by an erosion surface lined with endosteal bone (sb, electronic supplementary material, figure S2*b*). Erosion and endosteal ossification have thus already started operating on the internal face of this very young cortex (sb, electronic supplementary material, figure S2*c*), but some remnants of calcified cartilage (Liesegang rings and chondrocyte lacunae) are still present (respectively, Lr and cl, electronic supplementary material, figure S2*d*,*e*).

### (b) Adult humeri

**Figure 3.**
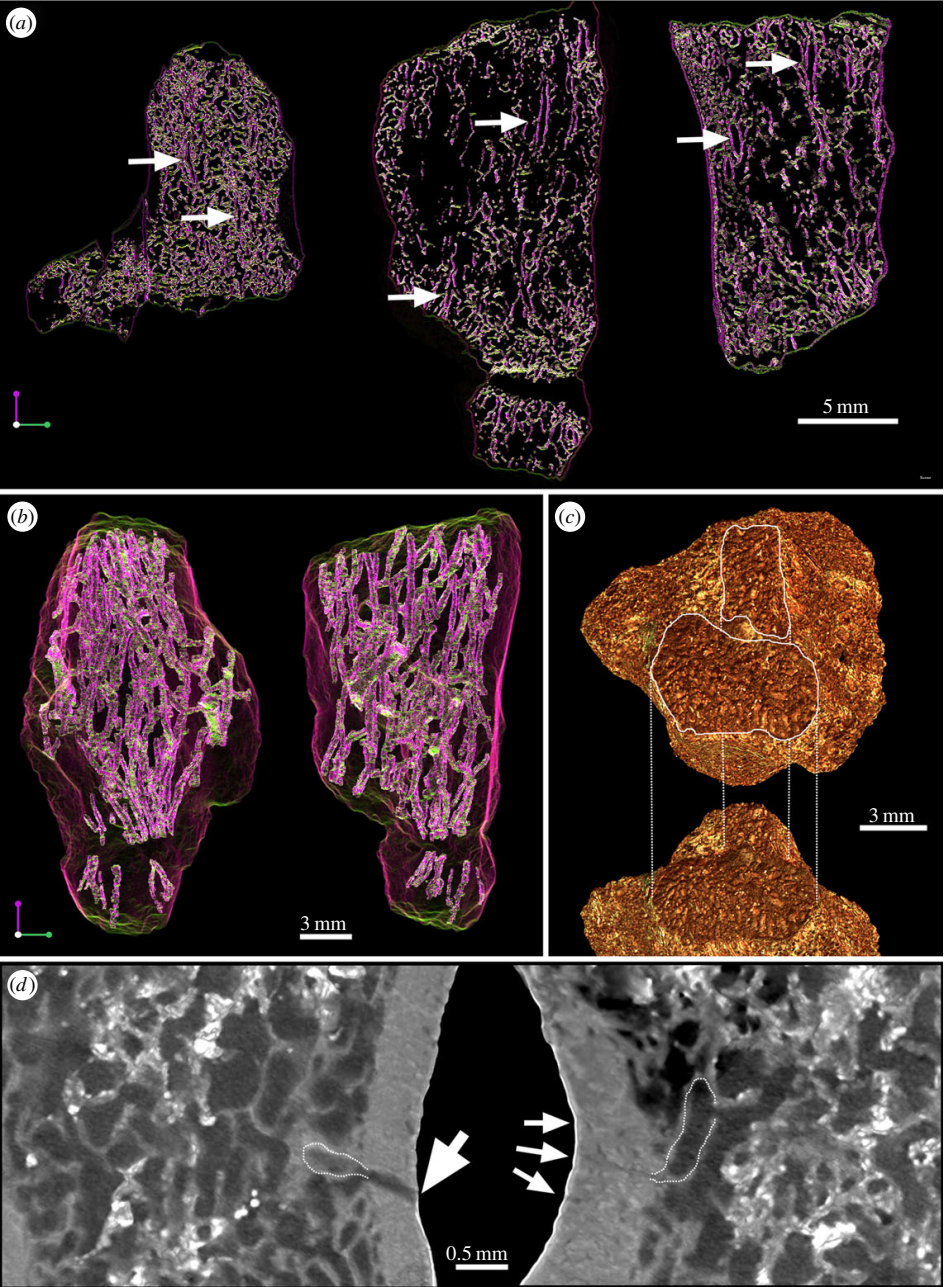
Organization of the spongiosa in the humerus of *Eusthenopteron*. (*a*) Virtual thin sections made in the same longitudinal plane in the three humeri of *Eusthenopteron* (from left to right: juvenile specimen NRM P246c, adult specimen NRM P248d, adult specimen NRM P248a). Based on a directional coloured light system, the longitudinal trabeculae appear in purple and the transverse trabeculae in green. The white arrows point out the longitudinal tubular structures. (*b*) Three-dimensional organization of the tubular structures within the spongiosa in mesial view and ventral view (adult *Eusthenopteron* NRM P248d). (*c*) Distal articular surfaces (adult *Eusthenopteron*, NRM P248d) showing a distinctive pattern of aborted channels, typical of the cartilage-bone junction in tetrapods, which produces bony septa in columns. (*d*) Images showing the connection of two tubular structures with the cortical vascular mesh (pointed out with white arrows) in the juvenile *Eusthenopteron* (NRM P246c).

At mid-shaft, the marrow spongiosa has spread to 96.5% of the total diameter due to internal erosion of the cortex, which now has an average thickness of only 290 µm (electronic supplementary material, figure S3*b*). Large bays of erosion, covered with a thin layer of endosteal bone (secondarily deposited and identified from the resorption line; figure 2*b*), cut into the compact bone layer. The boundary between the compacta and spongiosa therefore remains sharp (rl, figure 2*b*; electronic supplementary material, figure S3*b*). The spongiosa is less dense than in the juvenile and exhibits very thin endosteal trabeculae (electronic supplementary material, figures S1 and S3). It is dominated by longitudinal tubes, which cross the whole humerus between proximal and distal epiphyses (figure 3*a*,*b*; electronic supplementary material, figure S3). These tubes, 300 μm in diameter, end blindly at the articular surfaces of the epiphyses (figure 3*c*). They are anastomosed with smaller transverse tubules that fuse to the vascular mesh of the compact bone layer (figure 3*c*).

The mid-shaft cortical bone shows numerous small (typically 170–200 μm^3^), oval, homogeneously distributed osteocyte lacunae (figure 2*d*,*e*). The osteocyte lacunae of the endosteal bone are notably smaller, typically 50–100 μm^3^ (figure 2*d*). Average osteocyte volume is slightly smaller in the adult cortex than in the juvenile (electronic supplementary material, figure S4). Two lines of arrested growth (LAGs) are visible in the outermost region of the cortex (thick white arrows, figure 2*b*). The vascular mesh is mainly composed of radial canals, anastomosed at their bases with longitudinal small canals that parallel the inner surface of the cortex (v, figure 2*c*). The great majority of the radial blood vessels are closed off at the level of the first LAG; only a few reach the surface [21,27].

Towards the epiphysis, at the location of the ossification notch (electronic supplementary material, figure S5*a*) [24,28,29], the compact cortical bone tissue presents the same cellular and vascular organization as at mid-shaft (electronic supplementary material, figure S5*c*). The trabeculae in the spongiosa are greatly remodelled and show no visible remnant of calcified cartilage (electronic supplementary material, figure S5*b*).

### (c) Distal bones of adult fin

**Figure 4.**
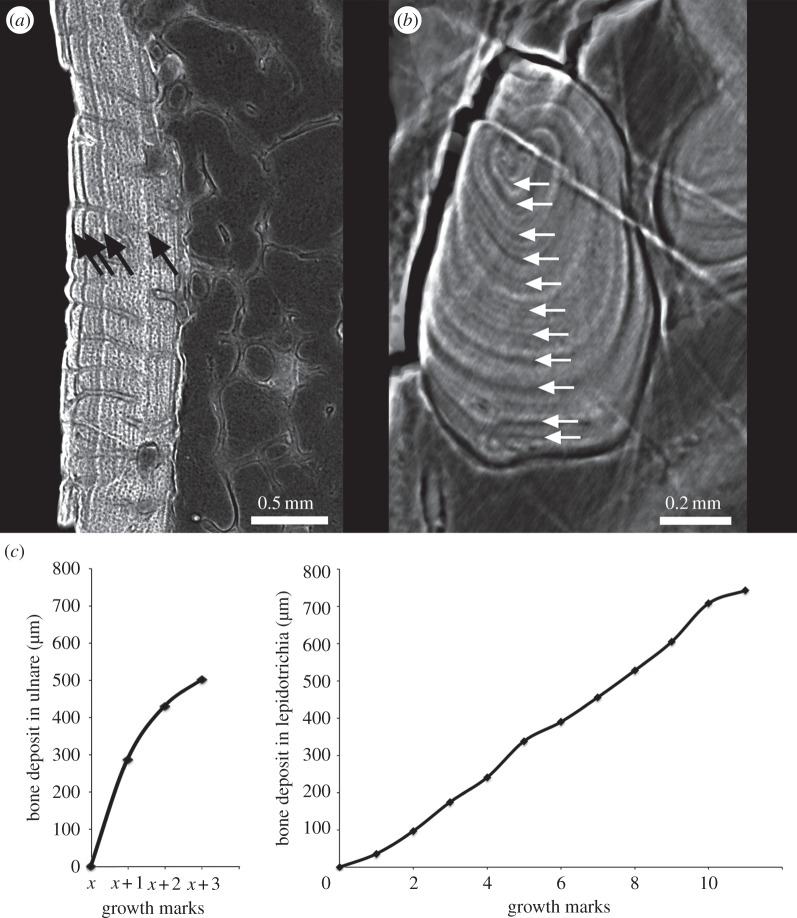
Skeletochronological analysis. (*a*) Longitudinal virtual thin sections made in the ulnare of *Eusthenopteron* (adult specimen NRM P248d) showing four LAGs (black arrows). Because the innermost region of the primary cortex was eroded, part of the LAG pattern is not visible anymore. A tightening of the peripheral LAGs is obvious. (*b*) Virtual thin section made in a basal lepidotrichia of the same adult specimen showing 11 LAGs (white arrows). The two last peripheral LAGs are slightly closer to each other. (*c*) Growth curves made from the measurement of the bone deposit accumulation of periosteal bone in the remaining cortex of the ulnare (electronic supplementary material, table S1) and in the lepidotrichia (electronic supplementary material, table S2).

The proximal end of the ulna of NRM P248d contains longitudinal tubes identical to those in the associated humerus (figure 3*b*). The associated ulnare, which also contains such tubes, has a proportionately thicker cortex than the humerus (figure 4*a*). Four LAGs are preserved, the last two much more closely spaced than the inner three. Despite its greater thickness, this cortex, like that of the humerus, shows resorption bays on its inner surface and has thus been subject to remodelling linked to medullary expansion.

Basal segments of lepidotrichia preserved in articulation with the fin endoskeleton contain 11 LAGs (figure 4*b*). These elements show no sign of internal resorption, and as they are known to ossify early in life in *Eusthenopteron* [30], they probably record the complete growth history of the specimen.

## 4. Discussion

### (a) A mosaic long-bone organization

In extant actinopterygians, the fin endoskeleton develops from a blastema that differentiates into cartilages [15]. Perichondral bone is deposited on the surface of the cartilages and continues to grow centrifugally as periosteal bone (electronic supplementary material, figure S6). Consequently, a typical metapterygial bone (electronic supplementary material, figure S6) is composed of a cartilaginous rod surrounded by a bony tube with cartilage projecting as condyles [15]. In older individuals, the rod of cartilage can be resorbed and sometimes replaced by endochondral ossification, resulting in a superficially ladder-like trabecular spongiosa [31,32] (electronic supplementary material, figure S6). Most of the space created by this process is filled with fatty tissue, nerves and blood vessels. There is no haematopoietic tissue [15,31].

In most extant tetrapods, by contrast, the cartilaginous rod only exists at an early stage of long-bone development [14]. It is rapidly covered with perichondral bone, and then periosteal bone, which thickens substantially in older individuals to form the cortical bone (electronic supplementary material, figure S7). At the articular extremities, an epiphyseal centre produces more cartilage, known as ‘growth cartilage’, consisting of longitudinal columns of aligned chondrocytes that become hypertrophied towards the diaphysis [33,34]. At mid-shaft, the cartilage template is progressively hollowed out, creating the medullary cavity [14,35]. When the erosion front reaches the base of the growth cartilage, some vascular channels and marrow processes begin to invade the columns of hypertrophied chondrocytes [34,36]. Endochondral bone is deposited along the cartilaginous septa of the growth plate and on the surface of remnants of cartilage in the diaphysis.

In the juvenile *Eusthenopteron*, the remnants of Katschenko's line at mid-shaft coupled with the large diameter of the medulla (approx. 5.3 mm, or 55% of the complete shaft diameter of the adult humerus; electronic supplementary material, figures S1 and S3) show that the cartilaginous humerus had grown quite large before the onset of ossification. This resembles the actinopterygian condition. However, the intricate trabecular architecture and presence of Liesegang rings of calcified cartilage in the juvenile are tetrapod-like characteristics. Similarly, the bone surfaces at the location of articulations exhibit a distinctive pattern of aborted channels resembling the cartilage-bone junction in most tetrapods [37–39]. As in non-amniotic tetrapods (and some amniotes), there was no secondary ossification centre [25,26,40] in the epiphyses of *Eusthenopteron*.

In summary, the humerus of *Eusthenopteron* shows a late onset of ossification similar to that seen in an extant actinopterygian, but the subsequent processes of cartilage resorption, endochondral ossification and elongation growth are all tetrapod-like. The single exception to this pattern is the strong internal resorption of the cortex and expansion of the medullary spongiosa revealed by adult humeri (electronic supplementary material, figure S3), which differs from both extant tetrapods and actinopterygians [16]. This may have served to maximize the volume of bone marrow (see below). The proportionally thicker cortical bone of more distal elements (e.g. ulnare, figure 4*a*; radius, ulna [16]) may reflect biomechanical requirements.

### (b) Growth curve and life history

Growth biology and metabolic features are recorded in long-bone cortical microstructure [41–44]. All the bones studied here come from the same locality (Miguasha, Canada) and show no evidence of any peculiar taphonomic degradation. A comparison between the microstructures of the juvenile and adult bones should thus yield reliable life-history data for *Eusthenopteron*.

The adult endoskeletal bones and lepidotrichia all show repeated LAGs indicating a cyclical growth pattern (figures 2 and 4). In the juvenile humerus, there are no visible LAGs, but cyclical variations in volume of the osteocyte lacunae reflect cyclical bone growth (cf. arrows pointing out recurrent decreases of bone cell volume, figure 1*d*). This is confirmed by small osteocyte lacunae at the location of LAGs in the adult (figure 2*b*,*d*; electronic supplementary material, figure S8). The cyclicity probably reflects an intrinsic biological cycle enhanced by environmental seasonality [28], which is annual in all extant tetrapods living in mild and warm seasonal climate conditions [28], like those of Miguasha during the Devonian [45]. In this case, the adult individual with preserved lepidotrichia was at least 11 years old at death (figure 4*c*). The last 2–3 years of life of the adult witnessed a dramatic slowing of growth, as evidenced in the ulnare and humerus by the prominent and closely spaced final two LAGs (figures 2*b* and 4*c*), the sealing off of the majority of cortical blood vessels by the first of these LAGs [21,27] and the greatly reduced osteocyte volume in the outermost bone layer (figure 2*d*; electronic supplementary material, figure S8).

Such a slow-down of bone deposition is well known in tetrapods [43,46] and probably reflects the onset of sexual maturity. In the lepidotrichia, which begin to ossify very early in life [30] and do not undergo internal resorption, 11 LAGs are present, and there is a notable slow-down between LAG 10 and 11 (figure 4*b*,*c*). These data thus suggest a pre-reproductive growth period of approximately 10–11 years for this individual of *Eusthenopteron*, which is considerably longer than in the majority of extant amphibians (pre-reproductive period of 5 years in average and rarely longer than 9 years for urodeles; 3 years in average for anurans) [28] and also longer than in the South American lungfish *Lepidosiren* (8 years maximum total life span; age of sexual maturity unknown) [47], but within the range of some sturgeons (e.g. shortnose sturgeon: pre-reproductive period of 2–11 years for males, 6–13 years for females, depending on population) [48] and slightly shorter than in the Australian lungfish *Neoceratodus* (pre-reproductive period of 15 years for males, 20 years for females) [49]. The size of the juvenile humerus of *Eusthenopteron* suggests that ossification of the pectoral fin endoskeleton began approximately halfway through this period. For comparison, the smallest complete individuals of *Eusthenopteron* from Miguasha with partly ossified humeri have a total body length of approximately 18.5–19 cm [30].

### (c) The earliest evidence for functional bone marrow

The humerus of *Eusthenopteron* is largely composed of cancellous bone. The organization of the trabecular mesh reflects a longitudinal tubular configuration that crosses the long bone from the proximal to the distal epiphyses (figure 3*a*,*b*). This longitudinal mesh is slightly transversally anastomosed and is connected to the cortical vascular canals (figure 3*d*), strongly suggesting that it had a role related to blood supply. The configuration resembles the arterial organization in the metaphyseal region of young tetrapod long bones [50,51].

In tetrapod epiphyses, during early development, the cartilage is organized in columns of chondrocytes separated by longitudinal bony septa [34]. Blood vessels progressively invade these cartilaginous columns and release diffusible factors that play an important role in the apoptosis of chondrocytes and the establishment of endochondral ossification at the chondro-osseus junction during elongation growth [34]. This process results in the longitudinal orientation of the metaphyseal trabecular mesh [40]. Not only vascular channels (10–30 μm in humans [36]) but also larger marrow processes (30–70 μm in humans [36]) penetrate the hypertrophied cartilage. In adult tetrapods, this longitudinal configuration can no longer be observed because intense erosion and remodelling incorporates the vascular mesh into the medullary cavity [52,53].

In contrast to tetrapods, actinopterygian long bones have no haematopoietic bone marrow but only fatty tissues in the spaces created by the erosion of the cartilaginous rod [15,54]. Neither do they show any evidence of a growth plate with longitudinally oriented columns of chondrocytes [15,40]. The large longitudinal tubular mesh observed in *Eusthenopteron* humerus appears to constitute the earliest and phylogenetically deepest documented occurrence of a complex functional bone marrow in the tetrapod stem group. As the tubular channels in *Eusthenopteron* are obviously connected to the epiphyses, the appearance of a complex bone marrow seems to be related to the appearance of tetrapod-like epiphyseal structures and elongation growth. *Eusthenopteron* lacks the comprehensive remodelling and trabecular resorption that creates an open medullary cavity in the majority of extant tetrapods, but the reduction of trabeculae between the longitudinal tubes in the adult compared with the juvenile may represent an evolutionary precursor of this process.

Bone marrow has mostly been studied for its haematopoietic properties [55–57]. However, the marrow is also a source of both osteoblasts and osteoclasts [58–60]. Tetrapod bone marrow has been shown capable of degrading cartilage proteoglycans and inducing the initial stage of endochondral ossification [61]. Cumulative evidence has shown strong links between osteoblasts and haematopoietic components in long bones of living tetrapods [62–64]. It seems that the establishment of certain haematopoietic niches is regulated by osteoblasts and/or osteoclasts, whose appearance precedes the activity of haematopoietic stem cells (HSCs) during development [57,63,65]. Some HSCs are functionally dependent on their proximity to endosteal surfaces [55], and HSC niches are frequently found close to the endosteal [51,66–68] or epiphyseal surfaces of bones [69]. The organization of the marrow space in *Eusthenopteron* argues for a functional link to extension growth at the epiphyses, suggesting that the intimate relationship between hypertrophic cartilage remodelling, endochondral ossification and haematopoiesis seen in extant mammals [38,39] is primitive for tetrapods. By contrast, the absence of haematopoietic niches in the limb bones of some amphibians [51] or birds can be interpreted as a secondary simplification of long bones with no endochondral trabeculae in amphibians [26,40] and pneumatization in birds [70].

## 5. Conclusion

*Eusthenopteron* proves to possess a more distinctive combination of biological and life-history traits than previously thought [17]. Morphologically, it is a conventional predatory ‘fish’ with no obvious terrestrial adaptations [30]. Its humerus is similar in relative size and proportions to those of other lobe-finned sarcopterygians [9]. Ossification of the humerus began when the element was more than half adult size and the animal apparently several years old, whereas extant tetrapod limb bones ossify much earlier [25,28,71]. *Eusthenopteron* humerus contains an organized spongiosa that seems to have housed a functional bone marrow, and it underwent tetrapod-like extension growth. A marked slowing of growth in the adult probably indicates the onset of sexual maturity. If this interpretation is correct, the pre-reproductive growth period spanned a whole decade, considerably longer than in extant amphibians [28]. Unlike either extant ‘fishes’ or amphibians, *Eusthenopteron* eroded the inner face of the humeral cortex so vigorously that it actually grew thinner, and the spongiosa more extensive, as the animal approached adulthood.

The morphology and phylogenetic position of *Eusthenopteron* show that its tetrapod-like humeral characteristics are not terrestrial adaptations, a point that is also underscored by the remarkable thinning of the humeral cortex, which must have lessened the mechanical strength of the adult bone. This raises important questions about the original functional significance of the emplacement of marrow into the limb bones and the adoption of tetrapod-like extension growth, as well as about their possible role as enabling factors for terrestrialization. In order to address these questions and investigate life-history evolution across the ‘fish–tetrapod transition’, more comparative histological and microanatomical data from long bones of other lobe-finned sarcopterygians and early tetrapods will be studied.
